# Efficacy of phloroglucinol and diclofenac sodium on pain relief for renal colic due to ureteral stones: a single-center, prospective randomized trial

**DOI:** 10.3389/fpain.2026.1877482

**Published:** 2026-07-16

**Authors:** Fangxin Gong, Guangfeng Shao, Xiulin Zhang, Mingzhen Yuan, Hanwen Liu

**Affiliations:** 1 Department of Urology, The Second Qilu Hospital of Shandong University, Jinan, Shandong, China; 2 Department of Urology, Shandong Provincial Hospital, Shandong University, Jinan, Shandong, China

**Keywords:** diclofenac sodium, phloroglucinol, prospective randomized trial, renal colic, ureteral stones

## Abstract

Renal colic is considered one of the most severe types of pain and requires prompt and effective analgesia. This study aimed to evaluate the efficacy of phloroglucinol in treating acute renal colic, comparing it to diclofenac sodium alone and in combination with phloroglucinol. In this single-center, randomized controlled trial, participants with acute renal colic (visual analog scale [VAS] score >4) due to ureterolithiasis were enrolled. They were randomly assigned (1:1:1) to receive diclofenac sodium, phloroglucinol, or a combination of both. The primary outcome was the pain relief rate at 30 min after treatment—defined as the proportion of participants with a ≥ 50% reduction in VAS score from baseline. Of 363 screened participants, 258 were enrolled and completed the trial (86 per group). The primary outcome was significantly better in the diclofenac sodium group than in the phloroglucinol group (55.8% vs. 70.9%; OR, 0.52; 95% CI, 0.28–0.97; *p* = 0.040). However, no significant difference was observed between the diclofenac group and the combination group (74.4% vs. 70.9%; OR, 1.19; 95% CI, 0.61–2.33; *p* = 0.608). Adverse events were significantly less frequent in the phloroglucinol group than in the other two groups. In conclusion, diclofenac sodium provides superior rapid pain relief and remains the optimal first-line treatment for acute renal colic. Overall, while it should not be used as a general alternative, phloroglucinol may be considered in patients in whom the use of NSAIDs is contraindicated.

**Clinical Trial Registration:** https://www.chictr.org.cn/showproj.html?proj=275717, ChiCTR2500104563.

## Introduction

Renal colic is a prevalent urological emergency, typically induced by ureteral stones, and is the most frequent symptom of ureterolithiasis (1–4). Often described as one of the most severe pains a patient can experience, it accounts for millions of emergency department visits worldwide (2). In recent years, its incidence has steadily increased (3). In 2019, over 15% of cases were reported in China, placing a significant financial burden on the healthcare system (5).

Pain relief is the primary goal in managing acute renal colic. Although various drugs are available, nonsteroidal anti-inflammatory drugs (NSAIDs) and opioids remain the primary therapy options (6). Previous studies have shown that NSAIDs offer superior analgesic effects compared to opioids and, importantly, are less likely to induce drug dependency, making them the first choice for acute renal colic management (3, 6). In China, intramuscular diclofenac sodium is widely used in patients with acute renal colic due to its ease of use and rapid absorption (7, 8). However, NSAIDs can induce adverse reactions such as gastric mucosal damage. In cases of acute obstruction, NSAIDs may interfere with the kidneys' autoregulatory mechanisms, thereby increasing the risk of renal failure (9, 10).

Since increased contractile activity of ureteral smooth muscle is the main cause of renal colic, spasmolytic drugs have demonstrated their utility in pain relief (11). In previous studies, drotaverine, a PDE4 inhibitor, is effective in relieving pain in more than two-thirds of patients with renal colic; papaverine, another spasmolytic agent, is as effective as diclofenac sodium in relieving acute renal colic (12, 13).

Phloroglucinol is an effective and well-tolerated antispasmodic agent, widely used for the treatment of biliary colic, renal colic, and gynecological spasmodic pain (14, 15). In China, intramuscular injection of phloroglucinol has been used for many years in the treatment of renal colic, either alone or in combination with NSAIDs (16). However, to our knowledge, there are still no clinical trials evaluating the efficacy of phloroglucinol alone in the treatment of acute renal colic. Moreover, clinical trials comparing the combination of phloroglucinol and NSAIDs with NSAIDs alone are inconclusive. Boubaker et al. found that the addition of phloroglucinol did not enhance the pain relief effects of piroxicam in acute renal colic (17). Conversely, Corvino et al. observed that phloroglucinol combined with dexketoprofen provided superior results compared to dexketoprofen alone in the treatment of acute renal colic (15). In light of these conflicting clinical evidence, our study aimed to provide definitive clinical evidence for the use of phloroglucinol in patients with acute renal colic. In this study, we evaluated the efficacy of phloroglucinol in the treatment of acute renal colic and compared it to diclofenac sodium alone as well as to their combination.

## Methods

### Study design and participants

This single-center, randomized controlled trial with three treatment groups was conducted at the Department of Urology of the Second Hospital of Shandong University. The study was approved by the ethics committee of the Second Hospital of Shandong University (approval number: KYLL2025327) and registered with the Chinese Clinical Trial Registry (registration number: ChiCTR2500104563) on June 19, 2025. All eligible participants gave their written informed consent before enrolling in the trial. The study was conducted per the Consolidated Standards of Reporting Trials (CONSORT) guidelines (18).

In this trial, we included male or female patients aged 18–65 years who were diagnosed with acute renal colic by CT scan or ultrasound (within the last 24 h) according to the guidelines of the European Association of Urology (6) and demonstrated moderate-to-severe renal colic with a visual analog scale (VAS) (19) score of ≥4 (range: 0–10, with higher scores indicating more severe pain). The exclusion criteria were the use of analgesics within 6 h prior to the visit; hypersensitivity to diclofenac sodium, phloroglucinol; history of asthma; congestive heart failure; renal or hepatic failure; peptic ulcer, pyloric obstruction, or intestinal obstruction; hematologic disorders such as hemophilia or coagulation disorders; thrombocytopenia (<50 × 10^9^/L); history of psychiatric disorders or substance abuse; and pregnancy or lactation.

### Randomization and masking

**Figure 1:**
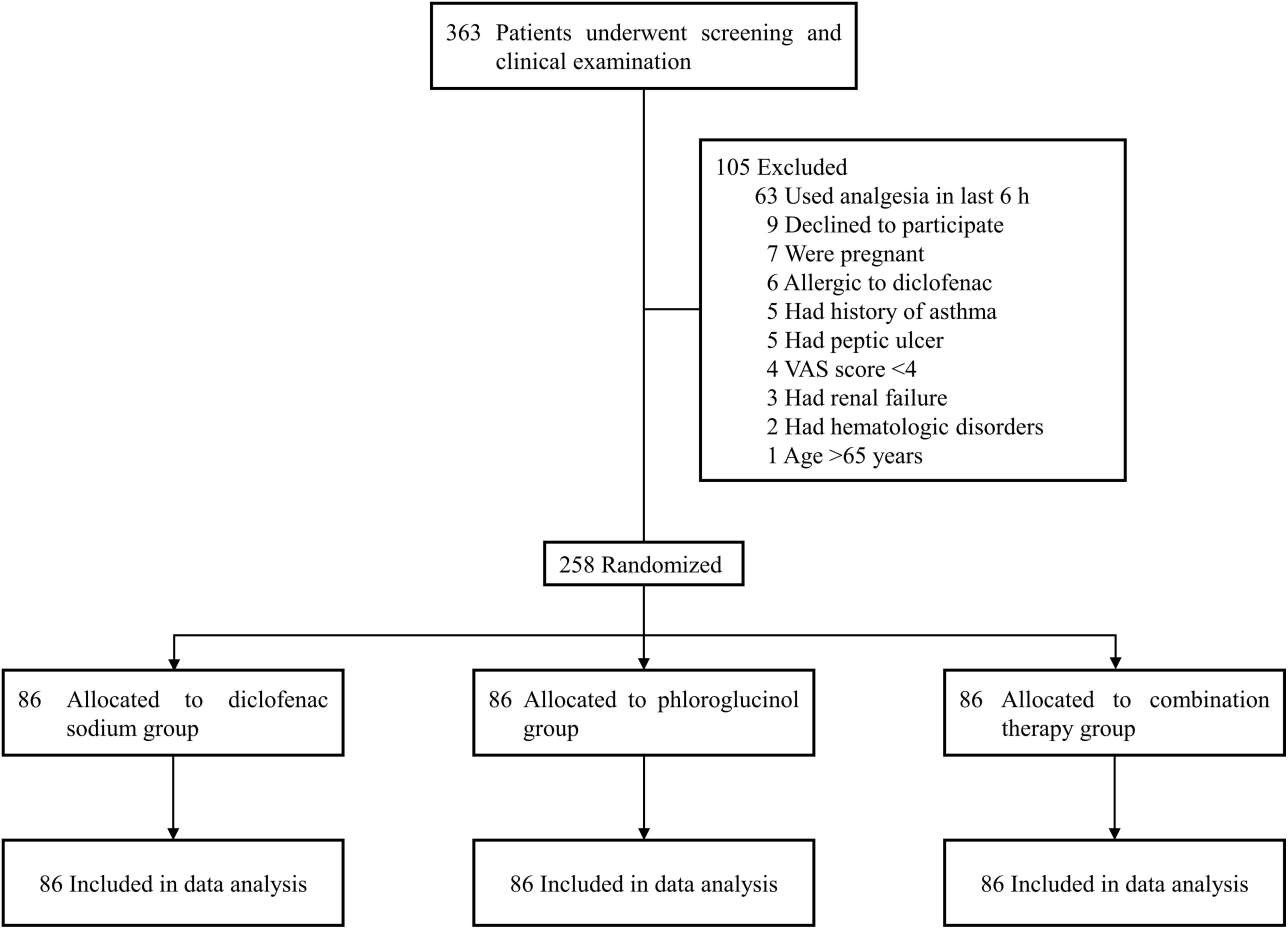
Trial profile. VAS indicates visual analog scale.

Participants were randomly assigned in a 1:1:1 ratio to receive either diclofenac sodium, phloroglucinol, or a combination of both. An independent statistician used an online tool to generate a blocked randomization sequence. Sealed envelopes were used to hide group assignments, which were kept by a research assistant who was not involved in recruitment, treatment, or evaluation. The envelopes were opened by the attending physician when eligible patients were enrolled in the trial. All other study personnel—including patients, outcome assessors, and statisticians—were blinded to the group partitioning. The patient enrollment flowchart is presented in Figure 1.

### Procedures

After the diagnosis of renal colic and randomization to groups, participants received 50 mg/2 mL of diclofenac sodium plus 1 mL normal saline intramuscularly, 40 mg/1 mL of phloroglucinol plus 2 mL normal saline intramuscularly, and combined treatment with 50 mg/2 mL of diclofenac sodium plus 40 mg/1 mL of phloroglucinol intramuscularly were administered in the appropriate groups.

Pain relief was assessed using the VAS, a commonly used pain intensity measure. The VAS consists of a 10 cm horizontal line, with endpoints labeled “no pain” (0) and “the highest possible pain” (10). The VAS score is determined by measuring the distance in centimeters from the left endpoint to the point marked by the participant, reflecting the severity of their pain.

No additional fluids were injected within 30 min following treatment. If the participant's expectation for pain relief was not met or the reported severity of pain was higher than 7 on the VAS at 30 min after treatment, rescue analgesia was employed until the VAS score consistently dropped to <5 or the participant refused further analgesia. The rescue analgesia in this study was 0.1 mg/kg intravenous injection of morphine sulfate. The vital signs of the patients were monitored during treatment. All adverse reactions were recorded.

All participants received equivalent treatment with the exception of the assigned medication. Participants were told to report colic recurrence via telephone after discharge from the hospital and were advised to visit the urology department. All participants were contacted by telephone 48 h after treatment to assess colic recurrence and readmission. In addition, all participants were contacted by telephone one week after treatment to assess the occurrence of adverse events.

### Outcomes

The primary outcome was the pain relief rate achieved 30 min after treatment. The pain relief rate is defined as the proportion of participants whose VAS score reduced by ≥50% compared to baseline.

Secondary outcomes included the pain relief rates at 60 min and 90 min after treatment; the VAS scores at 30, 60, and 90 min after treatment; the proportion of participants requiring rescue analgesia at 30 min after treatment; the proportion of participants with adverse events during acute management (within 90 min of treatment); and the rates of revisits and admission within 48 h of treatment, as well as the occurrence of adverse events at one week. Revisit and admission rates, along with adverse reactions, were assessed by trained investigators who contacted participants by telephone.

### Statistical analysis

The sample size was calculated based on previous literature and our clinical experience (3, 8). The pain relief rate at 30 min after treatment was expected to be 20% higher in the diclofenac sodium group than in the phloroglucinol group (70% vs. 50%). For 80% power and a type I error rate of 5%, we required 234 participants. We also accounted for an overall 10% loss to follow-up, resulting in a required sample size of 258 participants (86 in each group).

For baseline characteristics, continuous variables were described using the mean (standard deviation, SD), while categorical variables were presented as frequencies and percentages. Our statistical analysis was based on the intention-to-treat principle, including all randomly assigned participants. For each treatment, results were further expressed as odds ratio [OR], with its corresponding 95% CI, relative to the diclofenac sodium group. The one-way analysis of variance was used to compare normally distributed continuous variables among the three groups. Categorical variables were compared among the groups using the *χ*2 test or Fisher's exact test. The Kruskal–Wallis test was used to compare non-normally distributed continuous data among the groups. For *post-hoc* multiple comparisons among the three treatment groups, a Bonferroni correction was applied to control the family-wise Type I error rate.

To address the statistically significant difference in baseline VAS scores among the groups, a multivariable binary logistic regression model was utilized for the primary outcome. The treatment group and baseline VAS score were entered into the model to calculate the adjusted odds ratio (aOR) and 95% CI. Furthermore, to account for the confounding effect of rescue medication on delayed efficacy outcomes, a prespecified sensitivity analysis was conducted by excluding participants who received rescue analgesia, with the outcomes re-evaluated using unadjusted ORs, 95% CIs, and Chi-square tests.

All analyses were performed with SPSS version 27.0 (IBM), A two-sided *p* value of less than 0.05 was considered statistically significant.

## Results

Between June 2025 and October 2025, a total of 363 participants were screened, of whom 258 were enrolled in the trial and 105 were excluded (Figure 1). Enrolled participants were randomly assigned to one of three groups: diclofenac sodium (*n* = 86), phloroglucinol (*n* = 86), or combination therapy (*n* = 86). All participants completed the trial. Baseline characteristics were comparable across the three groups, as shown in Table 1.

**Table 1:** Baseline characteristics.

Characteristics	Diclofenac sodium (*n* = 86)	Phloroglucinol (*n* = 86)	Combination therapy (*n* = 86)
Demographics			
Age (years)	37.2 (9.2)	35.5 (8.5)	34.9 (9.5)
Sex			
Female	13 (15.1%)	16 (18.6%)	15 (17.4%)
Male	73 (84.9%)	70 (81.4%)	71 (82.6%)
Height (cm)	174.7 (5.9)	172.7 (7.0)	172.9 (7.5)
Weight (kg)	73.8 (12.9)	70.7 (13.3)	73.6 (13.5)
History			
History of previous urolithiasis	26 (30.2%)	20 (23.3%)	23 (26.7%)
Chronic illness			
Hypertension	23 (26.7%)	22 (25.6%)	18 (20.9%)
Diabetes	9 (10.5%)	8 (9.3%)	6 (7.0%)
Hyperlipemia	10 (11.6%)	13 (15.1%)	12 (14.0%)
Examination			
Pulse rate (per min)	80.4 (4.5)	79.5 (5.6)	78.4 (5.1)
Temperature (°C)	36.6 (0.2)	36.7 (0.2)	36.7 (0.2)
Systolic blood pressure (mmHg)	136.5 (8.0)	136.8 (8.0)	137.4 (7.8)
Diastolic blood pressure (mmHg)	83.7 (5.4)	83.4 (5.8)	84.8 (5.8)
Investigations			
White blood cell count (10^3^/μL)	10.0 (3.0)	10.2 (3.2)	10.3 (2.6)
Creatinine (mg/dL)	0.9 (0.2)	1.0 (0.2)	1.0 (0.2)
Stone size (mm)			
≤5	58 (67.4%)	55 (64.0%)	60 (69.8%)
>5	28 (32.6%)	31 (36.0%)	26 (30.2%)

Data are *n* (%), or mean (SD).

For the primary outcome, there was a statistically significant difference among the three groups (*p* = 0.022). Compared to the diclofenac sodium group, the phloroglucinol group had a significantly lower pain relief rate (55.8% vs. 70.9%; OR, 0.52; 95% CI, 0.28–0.97; *p* = 0.040). However, as reflected by the upper bound of the 95% CI approaching 1.0, the margin of this statistical superiority is relatively thin. To control for the potential confounding effect of the baseline imbalance in pain intensity, a multivariable binary logistic regression analysis was performed. After strictly adjusting for baseline VAS scores, diclofenac sodium remained significantly superior to phloroglucinol [adjusted OR (aOR), 0.45; 95% CI, 0.23–0.86; *p* = 0.016]. The combination therapy group showed an unadjusted pain relief rate of 74.4%; however, the adjusted analysis confirmed no significant difference in pain relief between the combination therapy and diclofenac sodium groups (aOR, 1.27; 95% CI, 0.64–2.52; *p* = 0.493).

**Figure 2:**
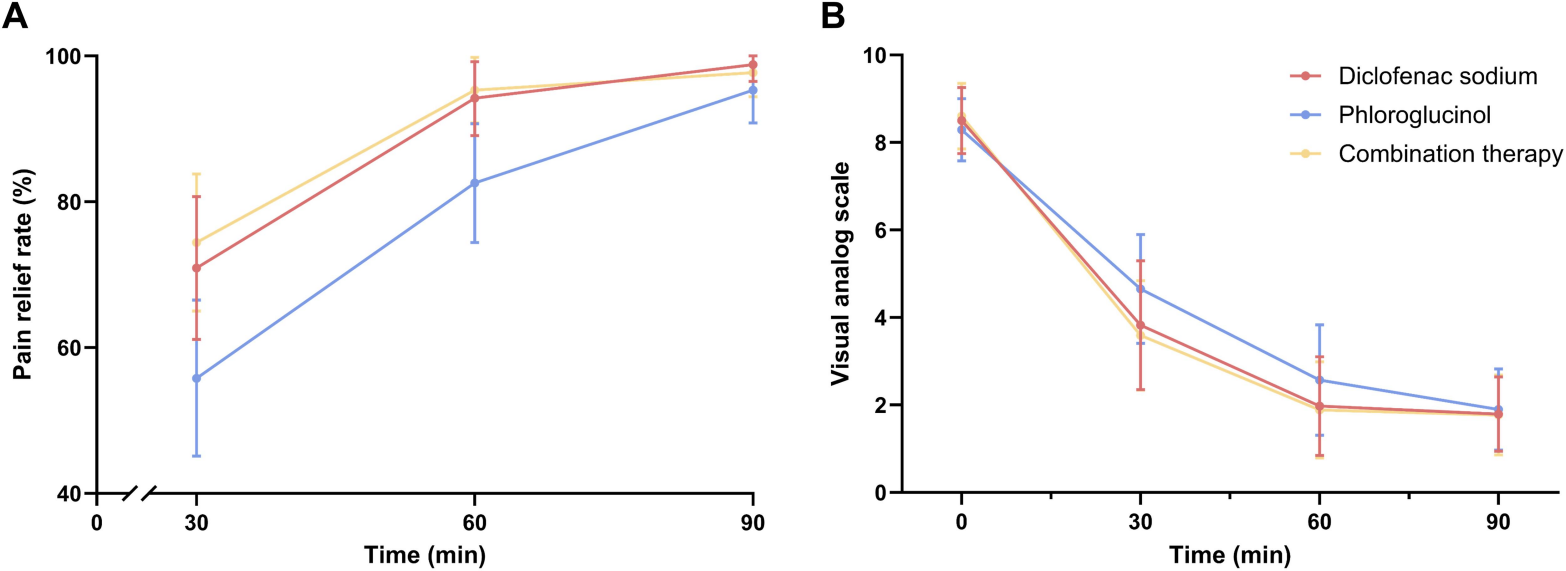
Trajectory of pain relief rate **(A)** and visual analog scale (VAS) scores **(B)**. Pain relief rate is defined as the proportion of participants whose VAS score reduced by at least 50% compared to baseline.

Comparing pain relief rates at 60 min, the diclofenac sodium group was also significantly better than the phloroglucinol group (82.6% vs. 94.2%; OR, 0.29; 95% CI, 0.10–0.84; *p* = 0.017), while no significant difference was observed between the combination therapy and diclofenac sodium groups (95.3% vs. 94.2%; OR, 1.27; 95% CI, 0.33–4.88; *p* = 0.732). Notably, at 90 min, the pain relief rates were nearly identical across all three groups. Table 2 and Figure 2 show the changes in pain relief rates over time.

**Table 2:** Primary and secondary outcomes.

Outcomes	Diclofenac sodium (*n* = 86)	Phloroglucinol (*n* = 86)	Combination therapy (*n* = 86)	*p* value
Pain relief rate^a^				
30 min^b^	61 (70.9%)	48 (55.8%)	64 (74.4%)	0.022
60 min	81 (94.2%)	71 (82.6%)	82 (95.3%)	0.006
90 min	85 (98.8%)	82 (95.3%)	84 (97.7%)	0.358
VAS score				
0 min	8.5 (0.8)	8.3 (0.7)	8.6 (0.8)	0.007
30 min	3.8 (1.5)	4.7 (1.2)	3.6 (1.3)	<0.001
60 min	2.0 (1.1)	2.6 (1.3)	1.9 (1.1)	<0.001
90 min	1.8 (0.9)	1.9 (0.9)	1.8 (0.9)	0.685
Rescue analgesia rate	10 (11.6%)	16 (18.6%)	8 (9.3%)	0.172
Acute adverse events	9 (10.5%)	0 (0%)	13 (15.1%)	<0.001
Nausea	3	0	5	
Vomiting	2	0	3	
Dizziness	3	0	4	
Rash	1	0	1	
Revisit and admission rate	11 (12.8%)	13 (15.1%)	16 (18.6%)	0.570

Data are *n* (%), or mean (SD).

a Pain relief rate is defined as the proportion of participants whose VAS score reduced by at least 50% compared to baseline.

b Pain relief rate at 30 min was the primary outcome; pain relief rates at other time points, VAS, rescue analgesia rate, acute adverse events, and revisit and admission rate were secondary outcomes.

Table 2 shows the VAS score at different time points among the groups. Compared with the phloroglucinol group, the diclofenac sodium and combination therapy groups demonstrated lower VAS scores at 30 min and 60 min. At 90 min, the VAS scores were similar across all three groups (Figure 2). Rescue analgesia was used by a significantly lower number of participants in the diclofenac sodium group compared to the phloroglucinol group (11.6% vs. 18.6%; OR, 1.74; 95% CI, 0.74–4.08; *p* = 0.202), whereas no statistically significant difference was detected in the combination therapy group (11.6% vs. 9.3%; OR, 0.78; 95% CI, 0.29–2.08; *p* = 0.618). To address the potential confounding effect of rescue analgesia, a prespecified sensitivity analysis was conducted by excluding participants who received rescue morphine. Among the participants who did not require rescue analgesia, the primary outcome (pain relief rate at 30 min) remained significantly higher in the diclofenac sodium group than in the phloroglucinol group (80.3% vs. 68.6%; OR, 0.54; 95% CI, 0.25-1.14; p=0.11). Furthermore, at 90 min, after removing the confounding effect of morphine, the pain relief rates reached 100% in both the diclofenac sodium and phloroglucinol groups, indicating identical delayed efficacy among the remaining participants without the need for further statistical testing.

There were no significant differences between the three groups in terms of revisit or admission rates. However, the overall incidence of acute adverse events was significantly higher in the diclofenac sodium group than in the phloroglucinol group (10.5% vs. 0%; OR, 0.47; 95% CI, 0.40–0.56; *p* = 0.002), with no difference observed compared to the combination therapy group (10.5% vs. 15.1%; OR, 1.52; 95% CI, 0.61–3.78; *p* = 0.361). Specifically, the 9 adverse events in the diclofenac sodium group included nausea (*n* = 3), vomiting (*n* = 2), dizziness (*n* = 3), and rash (*n* = 1). Similarly, the 13 events in the combination therapy group consisted of nausea (*n* = 5), vomiting (*n* = 3), dizziness (*n* = 4), and rash (*n* = 1). All reported acute adverse events were mild and transient. At follow-up after two weeks, no participants required hospitalization due to serious complications such as gastrointestinal bleeding, thrombocytopenia, or hepatic and renal failure.

## Discussion

In this randomized clinical trial, among patients with renal colic due to ureterolithiasis, the combination of diclofenac sodium and phloroglucinol showed similar efficacy to diclofenac sodium alone. This suggests that adding a spasmolytic agent to an NSAID may not enhance pain relief, consistent with findings from previous studies (13, 17, 20).

Our primary finding demonstrates that diclofenac sodium is statistically and clinically superior to phloroglucinol for rapid pain relief at 30 min (70.9% vs. 55.8%). Nevertheless, the thin margin of the 95% CI (0.28–0.97) indicates that this superiority, while significant, should not be overstated. For the general population, this advantage confirms diclofenac as the optimal first-line agent, and our results do not support the therapeutic equivalence of the two drugs. However, the clinical relevance of the 55.8% relief rate achieved by phloroglucinol should be interpreted within a stratified context. While it is less effective rapidly, achieving this degree of analgesia with a 0% incidence of acute adverse events is highly valuable for vulnerable populations. Therefore, rather than being a broadly optional alternative, phloroglucinol should be specifically considered in patients in whom the use of NSAIDs is strictly contraindicated.

Compared to phloroglucinol, diclofenac sodium provides more effective and rapid pain relief. Prostanoids such as prostaglandins (PGs), thromboxanes, and prostacyclins, mediate ureter contractions, increased pressure, and pain during ureteral obstruction (21). By blocking cyclooxygenase (COX), NSAIDs mitigate inflammation and profoundly suppress ureteral tension and spasticity. Nørregaard et al. found that parecoxib, a COX-2 inhibitor, reduced pelvic pressure in mice with ureteral obstruction (22). The inhibition effect of COX inhibitors on ureteral contraction was also confirmed by isolated ureteral muscle strip experiments. This study also showed that non-selective COX inhibitors and selective COX-2 inhibitors could suppress spontaneous contractions of human ureters, as well as spasmodic contractions evoked by electrical stimulation, high potassium, and Bay K 8,644 (21, 23, 24). Importantly, although phloroglucinol induces direct smooth muscle relaxation through a distinct pathway, both mechanisms ultimately converge to decrease intracellular calcium. The robust prostanoid inhibition by diclofenac likely maximizes ureteral relaxation, creating a “ceiling effect.” Consequently, adding phloroglucinol to this already saturated state provides no further synergistic benefit.

The proportion of participants requiring rescue analgesia was higher in the phloroglucinol group than in the diclofenac sodium group and the combination therapy group, which may indicate a more sustained pain relief from diclofenac sodium and is consistent with the results that phloroglucinol has a shorter half-life than diclofenac sodium (13, 15).

The incidence of acute adverse events with diclofenac sodium was higher than previously reported, which may be related to the age and gender composition of the study population, as well as differences in drug formulation technology (3). However, it is undeniable that NSAIDs, represented by diclofenac sodium, are not completely safe, and there are still many concerns regarding potential complications (25). While the incidence of adverse events is relatively low in healthy populations, NSAIDs pose significant risks for certain groups, such as pregnant women and the elderly. NSAIDs increase the risk of spontaneous abortion during pregnancy and are strongly associated with the development of neurodevelopmental, cardiovascular, respiratory and reproductive defects (26–28). The risk of cardiovascular, gastrointestinal, renal, and hepatic complications is higher with NSAIDs in the elderly (10). NSAIDs also increase the risk of renal failure in cases of acute obstruction due to ureteral stones (9).

To the best of our knowledge, there are no serious side effects associated with phloroglucinol, and no such events have been reported so far. In our study, there were no acute adverse events in the phloroglucinol group. The safety profile of phloroglucinol has been widely recognized, and in a study conducted in China, it was identified as the most commonly used by Chinese urologists for the treatment of renal colic in pregnancy (29).

### Limitations

This study has several limitations. First, the single-center design and lack of a placebo control limit generalizability and the assessment of true pharmacological effects versus natural disease resolution. Second, excluding patients with NSAID contraindications (e.g., peptic ulcers, renal impairment, asthma) yielded a young (aged 18–65), healthy cohort. Caution is needed when extrapolating results to elderly or comorbid patients who would benefit most from phloroglucinol's safety. Third, although baseline VAS scores differed significantly, our multivariable logistic regression strictly adjusted for this imbalance and confirmed it did not alter primary outcomes. Fourth, we could not perform subgroup analyses stratified by stone size or location. Stone locations were not systematically recorded, and subgroups for stones > 5 mm were statistically underpowered. Consequently, our results reflect an average response, restricting our ability to provide tailored, precision-medicine recommendations for specific stone characteristics. Finally, adverse events were partially evaluated via telephone interviews, potentially introducing detection bias, and the short two-week follow-up is insufficient to capture delayed complications or colic recurrence patterns.

## Conclusions

The results of this study indicate that intramuscular injection of either diclofenac sodium or phloroglucinol can rapidly relieve pain, with diclofenac sodium being the more effective option. However, phloroglucinol is associated with a better safety profile. Furthermore, the combination of diclofenac sodium and phloroglucinol does not offer superior pain relief. Overall, phloroglucinol may be considered in patients in whom the use of NSAIDs is contraindicated.

## Data Availability

The original contributions presented in the study are included in the article/Supplementary Material, further inquiries can be directed to the corresponding authors.
